# Supplementary material for: Genome-wide CRISPR Screens in T Helper Cells Reveal Pervasive Crosstalk between Activation and Differentiation
Source: Cell. 2019 Feb 7;176(4):882–896.e18. doi: 10.1016/j.cell.2018.11.044 (PMC6370901; doi:10.1016/j.cell.2018.11.044)
Supplement: Data S2. Processed Data from All the Steps of the Analysis, Related to Figure 1 [file mmc2.zip › supplemental data/motif analysis/Stat6_SRX021632_homer/homerResults/motif3.similar.html]

motif3

## Information for motif3

C
T
G
A
T
A
G
C
T
G
A
C
T
C
A
G
T
C
A
G
T
G
C
A
C
G
T
A
T
C
A
G
G
A
C
T
A
T
C
G
  
Reverse Opposite:  

T
A
G
C
C
T
G
A
A
G
T
C
G
C
A
T
A
C
G
T
A
G
T
C
A
G
T
C
A
C
T
G
A
T
C
G
G
A
C
T
  

|  |  |
| --- | --- |
| p-value: | 1e-1477 |
| log p-value: | -3.403e+03 |
| Information Content per bp: | 1.537 |
| Number of Target Sequences with motif | 8370.0 |
| Percentage of Target Sequences with motif | 33.11% |
| Number of Background Sequences with motif | 3129.9 |
| Percentage of Background Sequences with motif | 12.96% |
| Average Position of motif in Targets | 101.2 +/- 48.4bp |
| Average Position of motif in Background | 98.8 +/- 63.3bp |
| Strand Bias (log2 ratio + to - strand density) | -0.0 |
| Multiplicity (# of sites on avg that occur together) | 1.27 |
| Motif File: | file (matrix) reverse opposite |

### Similar de novo motifs found

|  |  |  |  |  |  |  |  |
| --- | --- | --- | --- | --- | --- | --- | --- |
| Rank | Match Score | Redundant Motif | P-value | log P-value | % of Targets | % of Background | Motif file |
| 1 | 0.976 | C T G A A G T C C G A T C G A T G A T C G T A C A C T G A T C G | 1e-1324 | -3049.146927 | 44.14% | 22.03% | motif file (matrix) |
| 2 | 0.934 | A G T C G A T C C T G A A G T C C G A T A C G T A T G C A G T C A C G T A T C G A G C T A G C T | 1e-1306 | -3007.404582 | 32.91% | 13.75% | motif file (matrix) |
| 3 | 0.606 | C G T A A G C T A C G T A G C T A G T C G A C T G A C T T C G A C T A G C T G A C G T A C T G A | 1e-1126 | -2593.344500 | 19.69% | 6.22% | motif file (matrix) |
| 4 | 0.604 | A C G T A C G T A G T C G A C T C G A T A T C G G C T A A C T G C G T A C G T A | 1e-856 | -1971.339103 | 13.07% | 3.64% | motif file (matrix) |
| 5 | 0.839 | C T G A A G T C C G T A A C T G A C T G C G T A C G T A C T A G | 1e-607 | -1398.521916 | 16.99% | 7.08% | motif file (matrix) |
| 6 | 0.846 | G A C T C G A T A G C T T A G C T A G C T C A G A G C T G A C T | 1e-384 | -885.434560 | 17.44% | 9.02% | motif file (matrix) |
| 7 | 0.750 | C T G A G A C T G C A T C G A T G A T C A G T C G A T C T C A G | 1e-234 | -540.391358 | 17.03% | 10.26% | motif file (matrix) |
| 8 | 0.735 | A G C T T G C A T A G C C T A G G A T C C T A G T A C G T G C A T C G A T C G A | 1e-181 | -419.029064 | 15.20% | 9.48% | motif file (matrix) |
